# Heat-induced Bone Diagenesis Probed by Vibrational Spectroscopy

**DOI:** 10.1038/s41598-018-34376-w

**Published:** 2018-10-29

**Authors:** M. P. M. Marques, A. P. Mamede, A. R. Vassalo, C. Makhoul, E. Cunha, D. Gonçalves, S. F. Parker, L. A. E. Batista de Carvalho

**Affiliations:** 1 Molecular Physical Chemistry R&D Unit, Department of Chemistry, University of Coimbra, Coimbra, Portugal; 2 Department of Life Sciences, University of Coimbra, Coimbra, Portugal; 3 Laboratory. Forensic Anthropology, Centre for Functional Ecology, University of Coimbra, Coimbra, Portugal; 4 Research Centre for Anthropology and Health (CIAS), University of Coimbra, Coimbra, Portugal; 5 Archaeosciences Laboratory., Directorate General Cultural Heritage (LARC/CIBIO/InBIO), Lisbon, Portugal; 6 ISIS Facility, STFC Rutherford Appleton Laboratory, Chilton, Didcot, OX 11 0QX United Kingdom

## Abstract

Complementary vibrational spectroscopic techniques – infrared, Raman and inelastic neutron scattering (INS) – were applied to the study of human bone burned under controlled conditions (400 to 1000 °C). This is an innovative way of tackling bone diagenesis upon burning, aiming at a quantitative evaluation of heat-induced dimensional changes allowing a reliable estimation of pre-burning skeletal dimensions. INS results allowed the concomitant observation of the hydroxyl libration (OH_libration_), hydroxyl stretching (ν(OH)) and (OH_libration_ + ν(OH)) combination modes, leading to an unambiguous assignment of these INS features to bioapatite and confirming hydroxylation of bone’s inorganic matrix. The OH_lib_, ν(OH) and ν_4_(PO_4_^3−^) bands were identified as spectral biomarkers, which displayed clear quantitative relationships with temperature revealing heat-induced changes in bone’s H-bonding pattern during the burning process. These results will enable the routine use of FTIR-ATR (Fourier Transform Infrared-Attenuated Total Reflectance) for the analysis of burned skeletal remains, which will be of the utmost significance in forensic, bioanthropological and archaeological contexts.

## Introduction

Bone is a biphasic material comprising a protein component (mostly collagen I) within an inorganic matrix of hydroxyapatite (Ca_10_(PO_4_)_6_OH_x_, HAp), the hydroxyl and phosphate groups being partly substituted by carbonate (respectively A- and B-type carbonates)^1,2^. Upon diagenesis – *post-mortem* processes triggering physical and chemical alterations – bone undergoes changes in molecular structure, namely recrystallisation (associated with collagen loss) leading to an increase in crystal size, carbonate depletion and uptake of anions from the environment (*e*.*g*. fluoride)^3–11^.

Human bones are often found in both archaeological and forensic contexts, and their importance for the study of past populations or for victim identification in forensic investigations is unquestionable (*e*.*g*. 9/11 terrorist attacks, 2009 Victoria bush fires). The examination of human skeletal remains may allow to establish biological profiles and thus contribute to the positive identification of the deceased. Also, it helps with inferring about the circumstances of death. However, bone vestiges can be found in diverse conditions and it is not unusual that the body has been subject to heat, upon events such as cremation, aircraft accidents, bush fires or acts of terrorism. In this case, to retrieve reliable information from the analysis of bones carries a high degree of uncertainty, because heat renders hydroxyapatite more accommodating to substitutions and often induces significant changes to the skeleton, which interferes with the reliability of the available osteometric methods^9,11–17^. Indeed, the techniques commonly used for human identification from burned bones and teeth are conceived for unmodified dimensions, while burning at high temperatures leads to considerable changes in the form of shrinkage or (more rarely) expansion. Overcoming this obstacle has been hindered by a scarcity of research on burned human remains, often for lack of accessible skeletons. This has had major implications, preventing anthropologists from being able to generate unequivocal biological profiles of both modern-day victims and individuals from past populations whose remains were recovered from archaeological settings.

Diagenesis-induced chemical alterations in bone (*e*.*g*. fluoridation, carbonate substitution or mineral uptake) can be accurately detected by optical vibrational spectroscopy (infrared and Raman). Additionally, Raman is the tool of choice for determining the presence of organic components, mainly collagen^18^, and has recently been used successfully for sub-surface probing of bone samples (through Spatially-Offset Raman spectroscopy (SORS))^19^. However, the marked fluorescence of bone (mainly assigned to its organic constituents) may limit the observation of the Raman signal^20^, particularly when the samples were not subject to high temperatures (>600 °C). Although this may be partially overcome by the use of near-infrared excitation (Fourier transform Raman, with a 1064 nm laser), Fourier transform infrared spectroscopy (FTIR) is nowadays the most commonly applied vibrational spectroscopic technique for these kinds of studies. A clear increase in recent years has been observed in its application within the forensic^15^ and archaeological^21–24^ sciences. For example, its potential for discriminating between fossilised, archaeological and modern bones^24–26^ or for estimating the postmortem interval in forensic cases^27–30^, has been demonstrated by evaluating bone crystallinity, as well as carbonate and organic contents. Nonetheless, devising a method capable of quantifying diagenetic changes in burned skeletal remains is still an unmet challenge. Although previous studies have attempted to tackle this issue by infrared spectroscopy^4,5,9–11,31–35^, an approach based on the intrinsic properties of bone (microcrystalline structure) was firstly followed by the authors through the application of inelastic neutron scattering (INS) spectroscopy to human bones burned under controlled conditions^36^.

The present study comes as a continuation of these promising results, using complementary vibrational spectroscopy techniques (both optical and neutron-based) with a view to attain, in the near future, a method that will enable us to extract quantitative information from burned human bones and estimate pre-burning skeletal dimensions in a reliable and consistent way. The main premise of that work is that the measured dimensional changes are primarily due to variations in the crystal structure of bioapatite, as heating is known to lead to an increased crystallite size and a decreased lattice strain (*i*.*e*. higher organisation). This is reflected in the crystallinity index (CI) and other spectroscopic ratios mainly obtained from infrared data^4,5,10,11^, which can then be used to assess the conditions of the burning process (*e*.*g*. temperature and duration) that affected the bones. Hence, a correlation will be sought between the pre-burning bone dimensions and the experimental set of data obtained for the burned samples: macroscopic heat-induced dimensional changes, and relevant spectroscopic biomarkers and indexes. The fact that variations in the elemental structure of bone are clearly reflected in its vibrational fingerprint fully justifies the use of vibrational spectroscopic methods to monitor bone diagenesis upon burning. Unlike optical techniques – Raman and FTIR^6,8–11,24,32,37,38^ – only a limited number of INS studies of bone have been carried out successfully in the last decade^36,39–41^. Nevertheless, these allowed detection of distinctive features such as changes in carbonate and water content, chemical substitutions at the hydroxyl sites and heat-elicited variations, revealing even minor differences in bone composition and being able to relate these to external factors affecting the skeletal samples (burning and/or other environmental events). INS is an extremely useful technique for probing a hydrogenous material such as bone, the intensity of each vibrational transition being expressed, for a given atom, by the dynamic structure factor

1 $${{\bf{S}}}_{{\bf{i}}}^{\ast }({\bf{Q}},\,{{\bf{v}}}_{{\bf{k}}})=\frac{({{\bf{Q}}}^{{\bf{2}}}{{\bf{u}}}_{{\bf{i}}}^{{\bf{2}}}){\rm{\sigma }}}{{\bf{3}}}{\bf{e}}{\bf{x}}{\bf{p}}(-\frac{{{\bf{Q}}}^{{\bf{2}}}{{\boldsymbol{\alpha }}}_{{\bf{i}}}^{{\bf{2}}}}{{\bf{3}}})$$

where $Q\,({\AA }^{-1})$ is the momentum transferred to the sample, *v*_**k**_ is the energy of a vibrational mode, ${{\rm{u}}}_{{\rm{i}}}(\AA )$ is the displacement vector of atom *i* in mode *k*, σ is the neutron scattering cross section of the atom, and ${\alpha }_{{\rm{i}}}(\AA )$ is related to a mass-weighted sum of the displacements of the atom in all vibrational modes. There are no selection rules for INS (in contrast to FTIR and Raman) which yields all the fundamental modes, overtones and combination bands for the analysed samples.

Complementary INS, Raman and FTIR (in attenuated total reflection mode, ATR) were applied to samples of human bone burned under controlled conditions (from 400 to 1000 °C). This is an innovative way of probing human bone tissue that will provide an improved understanding of heat-induced diagenesis and will lead to a quantitative method for assessing the associated structural and dimensional changes.

## Results and Discussion

**Figure 1:**
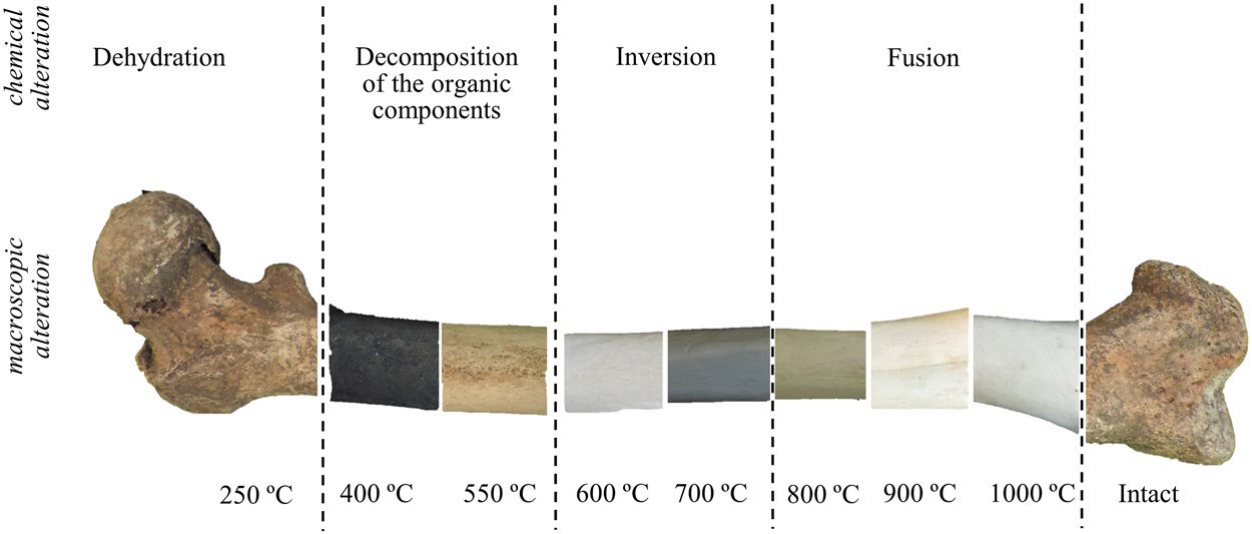
Schematic representation of the several stages of bone combustion.

The human skeletal remains currently analysed were burned under controlled laboratory conditions, at different temperatures (from 400 to 1000 °C). The process of bone combustion (burning under aerobic conditions) comprises several stages (Fig. 1)^12,42^: (i) dehydration – breakage of hydroxyl bonds and water removal (water molecules adhering to the hydroxyapatite crystals and bound to the organic constituents); (ii) decomposition of the organic components (lipids and proteins, at *ca*. 400 to 550 °C); (iii) inversion – carbonate loss upon heating (disappearing completely at *ca*. 700 °C); (iv) fusion (at >700 °C) – changes in crystallinity usually accompanied by an increase in crystal size, and OH^−^ and PO_4_^3−^ rearrangement within the pores left by the water and organic components. This thermal decomposition may be affected by the organic content of the bone, that acts as a shield against thermal damage protecting the inorganic component and thus leading to a delayed alteration of this mineral phase^43^. Thus, it is not until quite high temperatures are attained that the bone’s inorganic component is affected, microcrystallinity alterations taking place only above 700 °C. Actually, this temperature was found to be borderline, the most significant spectral changes having been detected above it (both by FTIR and INS), as discussed below.

In the present work, the application of complementary vibrational spectroscopic techniques (optical and neutron-based) delivered the whole vibrational profile of the bone samples under analysis: (i) the main bands ascribed to phosphate (PO_4_^3−^) and carbonate (CO_3_^2−^) groups, as well as to bone’s organic constituents (amide signals/proteins and CH_2_ deformation and stretching modes/lipids), obtained by Raman and FTIR; (ii) bioapatite’s OH librational and stretching bands, clearly detected by INS^36^ but not always seen in the optical spectra; (iii) the characteristic modes of the crystal lattice, associated with the short-range order and hydrogen-bonding profile within the crystalline framework (therefore reflecting bone’s heat-induced dimensional changes), that can only be accessed by neutron spectroscopy. Furthermore, the use of the MAPS and TOSCA INS spectrometers enabled the observation, with very high sensitivity, of both the low frequency region (<1000 cm^−1^, in TOSCA) and the high wavenumber range (in MAPS). Additionally, MAPS allowed the simultaneous detection of the OH librational and stretching signals from bioapatite.

### Infrared and Raman Spectroscopy

For the currently studied samples, infrared data was measured in reflectance mode (FTIR-ATR), covering a wide spectral window from the far- to the mid-IR (50 to 4000 cm^−1^), which has been shown to be a rapid, easy and cost-efficient method providing very reliable and reproducible results on bone, without any particular sample preparation and in a completely non-destructive way. In addition, these measurements have shown a higher reproducibility as compared to transmission FTIR (involving manual pellet creation) and other infrared methodologies such as diffuse reflectance infrared Fourier transform spectroscopy (DRIFTS, requiring sample mixing with KBr without pelleting)^8^.

Although the high fluorescence of bone, that restricts the observation of its Raman spectrum, can be slightly decreased by cleaning the bone fragments prior to measurement^44^, these are rather invasive procedures that were not applied in this work due to their potential interference in the bone’s characteristics, which we aimed to determine solely as a function of the heating temperature (for a constant burning time). Therefore, reliable Raman data was only obtained for bones subject to temperatures above 600 °C that yielded clearly observable bands.

Table 1 comprises the complete vibrational data (infrared, Raman and INS) measured for the human bones presently analysed and the corresponding assignments – both the high (>700 °C) and low temperature (<700 °C) vibrational signatures.

**Table 1:** Experimental (INS, FTIR and Raman) vibrational wavenumbers (cm^−1^) for the human bones analysed in this study (powdered samples), subject to different burning temperatures (in aerobic conditions)^a^.

<700 °C		Assignment	>700 °C		
FTIR-ATR	INS		FTIR-ATR	Raman	INS
		OH_lib_ + OH_stretching_			4250
		OH_stretching_	3570	3570	3570
3300	3385	OH_water stretching_			
2960–2850	2974	CH_stretching_			
	2560	3^rd^ overtone OH_libration_			2560
	1941	2^nd^ overtone OH_libration_			1941
1660	1660	amide I			
1650	1650	(H_2_O)_deformation_			
1550	1550	amide II			
1450	1450	(CH_2_)_deformation_			
1460–1415		ν_3_(CO_3_^2−^)	1460–1415		
	1302	1^st^ overtone OH_libration_			1302
1250 *w*	1250	amide III			
		ν_1_(CO_3_^2−^)_B_		1076	
		ν_3_(PO_4_^3−^)_fluorapatite_	1087	1028–1054	
1025		ν_3_(PO_4_^3−^)	1025		
		ν_1_(HPO_4_^2−^)	983 *sh*		
960		ν_1_(PO_4_^3−^)	960	960	
700 *w*		ν_4_(CO_3_^2−^)	700 *w*		
630	650	OH_libration_	630		638, 657
565, 605		ν_4_(PO_4_^3−^)	565, 605	578–617	574–617
470		ν_2_(PO_4_^3−^)/ν_2_(HPO_4_^2−^)	470	429–446	450
		OH_translation_	340	335	330–350
		ν(Ca–OH)_lattice_	330, 230 *sh*	329	330–350
	250	(CH_3_)_torsion_			
		ν(Ca– PO_4_)_lattice_	170, 210, 265	150, 200, 280	150, 190, 280
		ν(Ca–PO_4_)_lattice, translation_	85	138	70, 100

*sh –* shoulder; *w –* weak.

**Figure 2:**
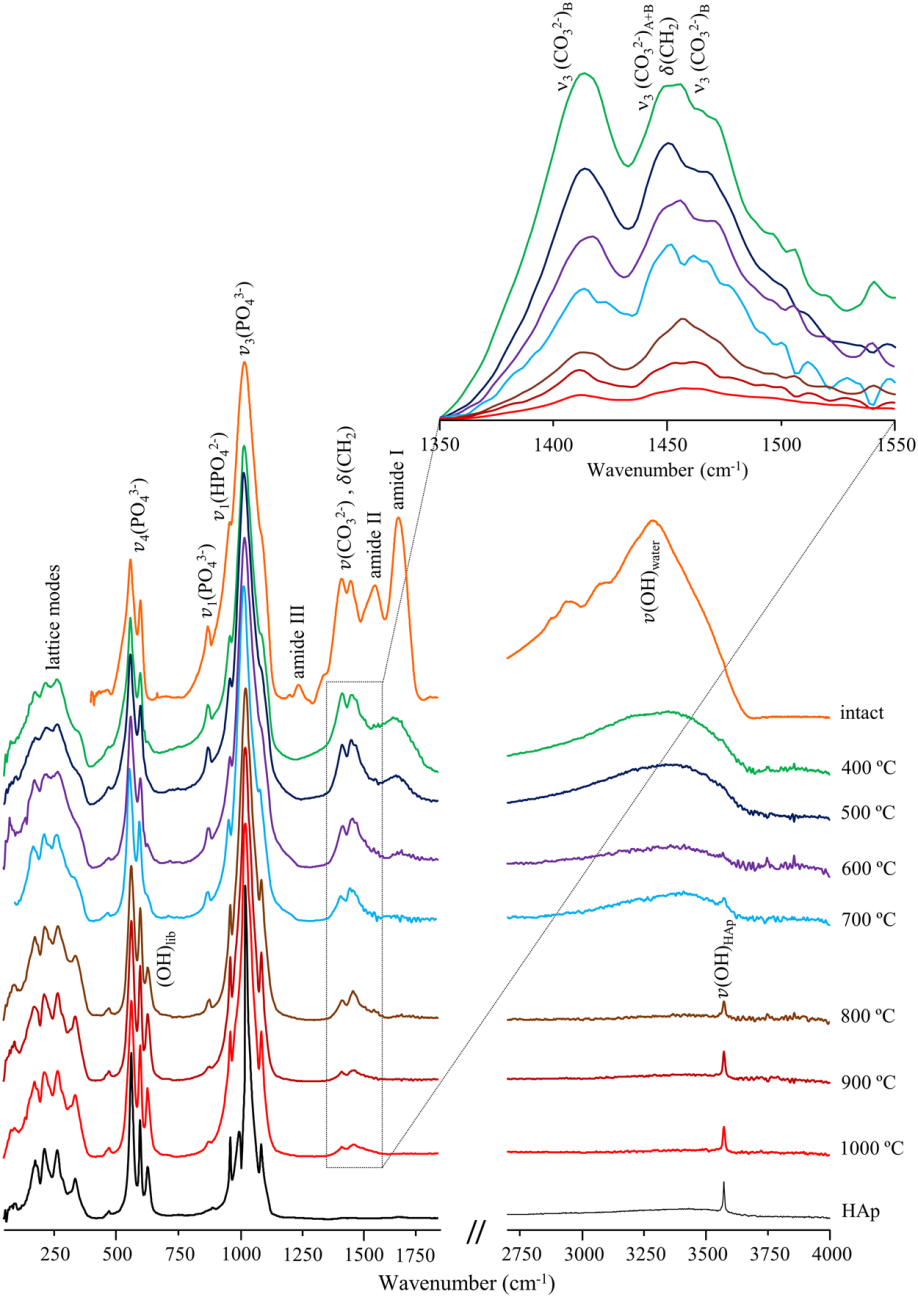
FTIR-ATR spectra (far- and mid-IR regions) of human humerus: intact and burned at different temperatures (400 to 1000 °C). The insert shows a magnification of the asymmetric stretching carbonate bands (ν_3_(CO_3_^2−^)). The spectrum of reference calcium hydroxyapatite (HAp, SRM 2910b) is also shown for comparison.

Figure 2 contains the FTIR-ATR spectra of human humeri, unburned and burned at defined temperatures from 400 to 1000 °C, as well as the infrared profile of reference hydroxyapatite (HAp). For the samples subject to temperatures below 700 °C, the infrared signature of bone’s organic constituents was clearly detected: from the lipids – a broad CH_2_ deformation band centred at 1450 cm^−1^ and a CH stretching feature at *ca*. 2950 cm^−1^ – and from the protein (mainly collagen type I) – amide I (1660 cm^−1^), amide II (1550 cm^−1^) and amide III (1250 cm^−1^). This organic matrix was completely destroyed above 700 °C, the lipidic constituents being the first to be removed, yielding a vibrational pattern very similar to the one formerly obtained for the same bone samples after defatting/deproteination procedures^45^.

The trigonal planar carbonate ions within bone yield distinctive signals at 1415/1460 cm^−1^ (ν_3_(CO_3_^2−^)). The band from ν_3_(PO_4_^3−^) dominates the spectra, at 1025 cm^−1^, surrounded by the features assigned to ν_1_(PO_4_^3−^) (960 cm^−1^) and ν_3_(PO_4_^3−^) from a second phosphate site (at 1087 cm^−1^, probably due to the presence of fluorapatite (francolite)), the latter being gradually obscured by the broadening of the ν_3_^as^(PO_4_^3−^) signal at temperatures below 600 °C. In addition, a shoulder is detected at *ca*. 983 cm^−1^ for the samples burned at 900 and 1000 °C, assigned to ν_1_(HPO_4_^2−^). The lower frequency range comprises the features ascribed to ν_4_(PO_4_^3−^) (565 and 605 cm^−1^) and ν_2_(PO_4_^3−^)/ν_2_(HPO_4_^2−^) (*ca*. 470 cm^−1^), as well as ν(Ca–OH) (*ca*. 330 and 230 cm^−1^), OH_translation_ (at *ca*. 340 cm^−1^) and (Ca–PO_4_) lattice modes (below 300 cm^−1^) from bone’s inorganic framework. The heat-induced reorganisation of bioapatite’s microcrystalline structure is revealed by the progressive narrowing of the bands upon increasing temperatures, coupled to the appearance of the characteristic signals from the hydroxyl groups at temperatures above 700 °C – (OH)_libration_ and (OH)_stretch_ at 630 and 3570 cm^−1^, respectively.

The vibrational profile currently observed for the bone carbonates deserves particular attention: the main detected features (at *ca*. 1415/1450/1460 cm^−1^) are ascribed to the ν_3_(CO_3_^2−^) asymmetric stretching (Table 1, Fig. 2), its threefold splitting being suggested to be due to the two distinct types of carbonate present in bioapatite. These display different orientations within the lattice, either perpendicular (type A) or parallel (type B) to the *c* axis, corresponding, respectively, to a CO_3_^2−^ substitution of either OH^−^ (in the channels) or PO_4_^3−^ (in the tetrahedral sites) within the apatitic structure^46–49^. These features undergo a marked decrease upon heating (>700 °C, Fig. 2), being almost absent in the samples burned at 1000 °C, as previously verified for carbonate apatites^49^, which is consistent with carbonate loss upon bone heating. This behaviour of the ν_3_(CO_3_^2−^) modes is in agreement with other reported work on the temperature dependence of the carbonate content of both mineral and skeletal samples. Owing to their distinct structures, carbonates A and B have been reported to display different thermal decomposition mechanisms, as they occupy sites within the inorganic network which are not equally affected by high temperatures^48,49^. Also, for synthetic calcium carbonate minerals it was found that ν_3_(CO_3_^2−^)_B_ is more responsive to external changes^50^ (such as heating), mainly due to structural differences within the bioapatite matrix (*e*.*g*. planar *vs* non-planar arrangements of the CO_3_^2−^ units). The bands currently observed at 1415 and 1460 cm^−1^ are ascribed to type B carbonates and were shown to be more prone to heat-induced removal, their intensity decreasing faster with increasing temperatures as compared to the signal at 1450 cm^−1^ assigned to carbonates A and B.

**Figure 3:**
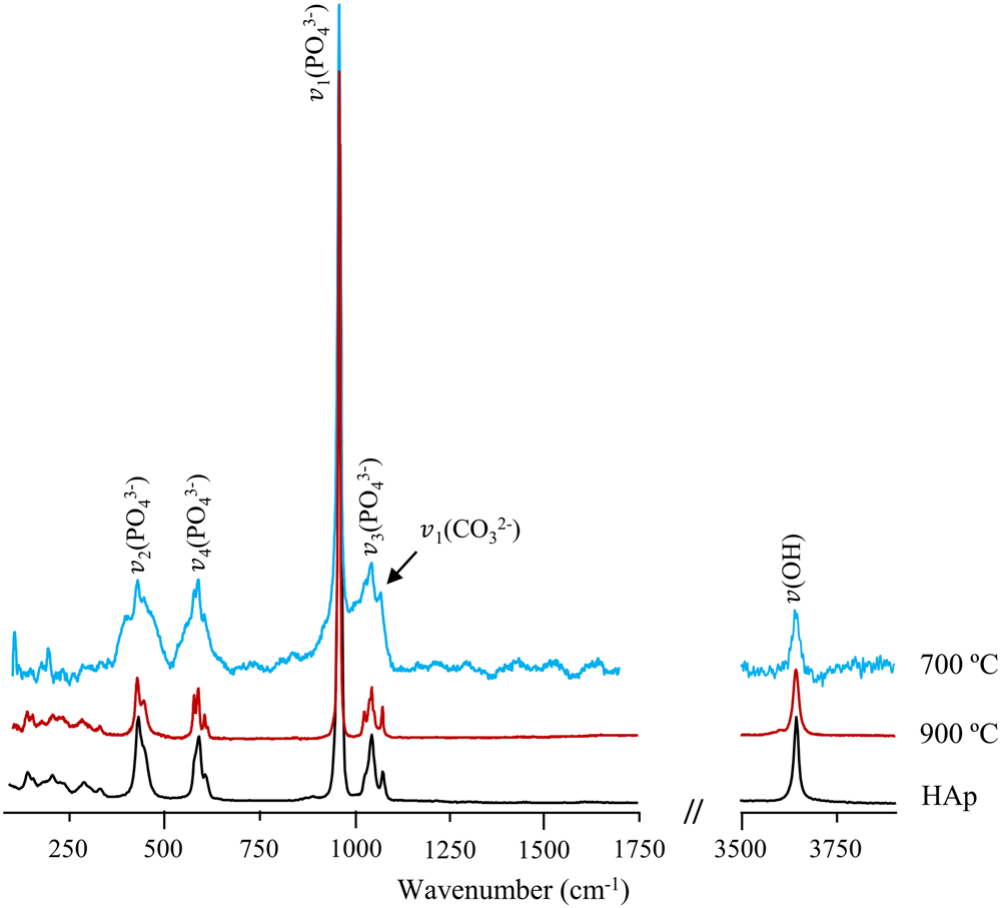
Raman spectra of human femur, burned at 700 and 900 °C. The spectrum of reference calcium hydroxyapatite (HAp, SRM 2910b) is also shown for comparison.

The complementary Raman results obtained for the bone samples presently studied (subject to temperatures above 700 °C) allowed access to vibrational modes not clearly detected by FTIR (Table 1, Fig. 3): (i) (Ca–CO_3_) lattice vibrations (*ca*. 140–280 cm^−1^), that did not show a particularly strong temperature dependence; (ii) phosphate modes – ν_2_(PO_4_^3−^) at 429/446 cm^−1^, ν_4_(PO_4_^3−^) from 578 to 617 cm^−1^, ν_1_(PO_4_^3−^) dominating the spectra at 960 cm^−1^, and ν_3_(PO_4_^3−^) at 1028–1054 cm^−1^; (iii) and a characteristic carbonate band at 1076 (ν_1_(CO_3_^2−^)_B_) (not observed in the infrared profile since it is obscured by the very intense ν_3_^as^(PO_4_^3−^) signal at 1025 cm^−1^). The broadening of the phosphate symmetric stretching (ν_1_(PO_4_^3−^)) for decreasing temperatures (900 to 700 °C) reflects a higher disorder of the bioapatite lattice, associated with a larger amount of CO_3_^2−^ ions (substituting either OH^−^ or PO_4_^3−^), since Raman spectroscopy is very sensitive to the short-range order within a unit cell. In addition, it should be emphasized that this variation in the bone’s carbonate content is closely related to alterations in the growth morphology and crystallite size, that are known to take place upon temperature changes – a higher atomic disorder corresponding to smaller crystal dimensions (in the nanometre scale), the lattice becoming progressively more ordered with increasing temperatures (upon CO_3_^2−^ loss).

### INS

High quality INS spectra were obtained for the burned samples, as they were free from the features assigned to the bone’s organic matrix (due to the protons from water, lipids and collagen)^51^, which were progressively destroyed by the burning process. This ensured a clear detection of the signals from hydroxyapatite, particularly the OH libration (centred at 650 cm^−1^), which reflects the H-bonding network within bone’s inorganic lattice. Interpretation of the data was assisted by previous results obtained for non-burned bovine and rat bones^39,40^ as well as by the first reported INS results on burned human bones^36^.

**Figure 4:**
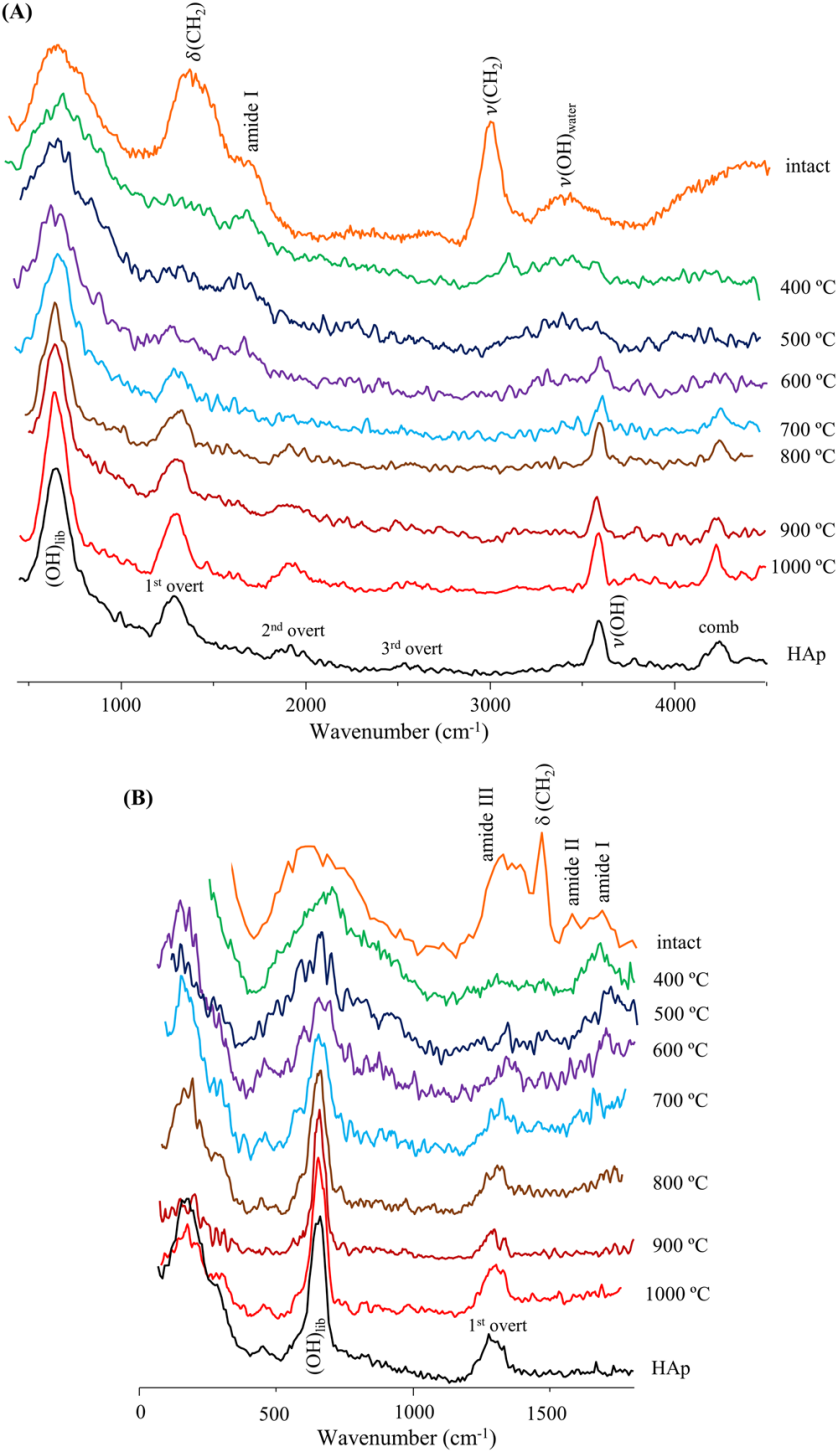
INS spectra, measured in MAPS, of human humerus: intact and burned at different temperatures (400 to 1000 °C). The spectra were recorded with 5240 (**A**) and 2024 cm^−1^ (**B**) incident energies. The spectrum of reference calcium hydroxyapatite (HAp, SRM 2910b) is also shown for comparison.

Special attention was paid to the bioapatite’s OH vibrational modes, prone to undergo marked changes upon heat-induced variations in the crystalline framework. For the samples subjected to very high temperatures (>800 °C) measured in MAPS, the OH libration and stretching signals were detected simultaneously (respectively at *ca*. 650 and 3570 cm^−1^) as well as the (OH_libration_ + OH_stretch_) combination band (at *ca*. 4250 cm^−1^) and the first, second and third overtones of the hydroxyl libration (at 1302, 1941 and 2560 cm^−1^) (Fig. 4). This concomitant observation of OH_stretch_ and (OH_libration_ + OH_stretch_)_comb_ allowed an irrefutable assignment of these INS features to bioapatite. Hence, bioapatite’s hydroxylation was definitely confirmed by these results (that corroborate those previously reported by the authors)^36^.

**Figure 5:**
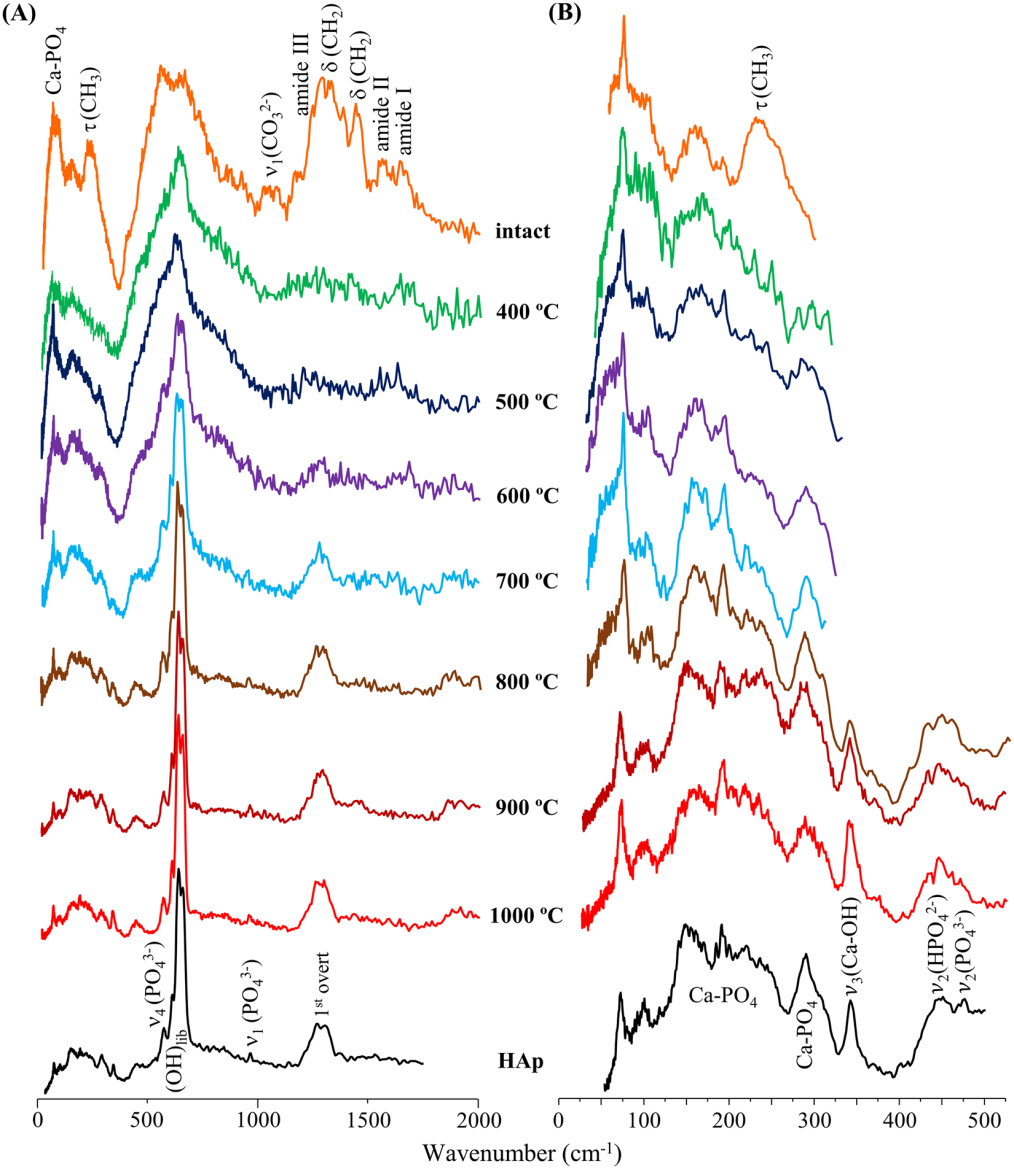
INS spectra, measured in TOSCA, of human femur: intact and burned at different temperatures (400 to 1000 °C). (**A**) 0 to 2000 cm^−1^; (**B**) 0 to 550 cm^−1^ (this region is expanded vertically for clarity sake). The spectrum of reference calcium hydroxyapatite (HAp, SRM 2910b) is also shown for comparison.

For the unburned bones, the spectra were dominated, as expected, by features from lipids and protein (mostly collagen I) at *ca*. 250 (protein methyl torsion), 1250 (amide III), 1450 (CH_2_ deformation), 1550 (amide II), 1660 (amide I) and 2974 cm^−1^ (CH stretching). These gradually disappeared upon heating: the lipidic components at *ca*. 500 °C, followed by the proteins that are hardly noticeable at 700 °C and completely destroyed at 900 °C. The main signals from the hydroxyapatite matrix were thus unveiled at temperatures above 700 °C (Figs 4 and 5).

An increased degree of crystallinity for higher burning temperatures (as previously reported)^40^ was evidenced by the noticeable narrowing of the INS signals, mainly the OH_libration_ band at 650 cm^−1^. Crystallinity reached a maximum at 900 °C, the corresponding spectral profile displaying a remarkable resemblance to that of the highly crystalline hydroxyapatite reference (SRM 2910b from NIST) (Figs 4 and 5).

The data recorded in TOSCA allowed access to the low energy range of the spectrum with very high sensitivity (Fig. 5), yielding relevant information on specific vibrational features particularly informative of the type of organization within the solid matrix^7,52^: (i) Ca^2+^ and PO_4_^3−^ sublattice translational and PO_4_^3−^ librational modes (below 300 cm^−1^), extremely sensitive to crystallinity and evidencing changes mainly between 700 and 800 °C; (ii) (Ca–OH) stretching and OH translation from hydroxyapatite (330–350 cm^−1^); (iii) ν_2_(HPO_4_^2−^) and ν_2_(PO_4_^3−^) (*ca*. 450 cm^−1^), the former being very difficult to detect by optical vibrational techniques; (iv) ν_4_(PO_4_^3−^) (574–617 cm^−1^), suggested to be due to distinct hydroxyapatite polymorphic species (hexagonal and monoclinic forms displaying very subtle and temperature-dependent structural differences); (v) splitting of the hydroxyapatite’s OH librational band (at 650 cm^−1^) due to the corresponding in-phase and out-of-phase modes (indistinguishable below 600 °C); (vi) CH_3_ torsion (at *ca*. 250 cm^−1^), detected for unburned bones due to the presence of protein. In particular, the ν_3_(Ca–OH) mode at 330 cm^−1^ was observed only for burning temperatures equal or above 700 °C, being absent for the lower temperatures (Fig. 5(B)), which is justified by the expected CO_3_^2−^ by OH substitution within the bioapatite network with increasing temperature (Table 2). In turn, the (Ca–CO_3_^2−^) vibrations (typically around 150 and 280 cm^−1^) were not clearly distinguishable below 700 °C, since they were hidden by the (Ca–PO_4_) lattice modes.

**Table 2:** Vibrational biomarkers of heat-induced diagenesis in human bone.

Vibrational band	wavenumber (cm^−1^)	detected by	heat-prompted variation
ν_3_(Ca–OH)	330	FTIR, INS	intensity decrease not detected <700 °C
ν_4_(PO_4_^3−^)	574–617	FTIR, INS	shift to higher frequency (600–1000 °C) not detected <600 °C
(OH)_lib_	650	FTIR, INS	shift to lower frequency
ν(OH)	3570	FTIR, Raman, INS	shift to higher frequency

**Figure 6:**
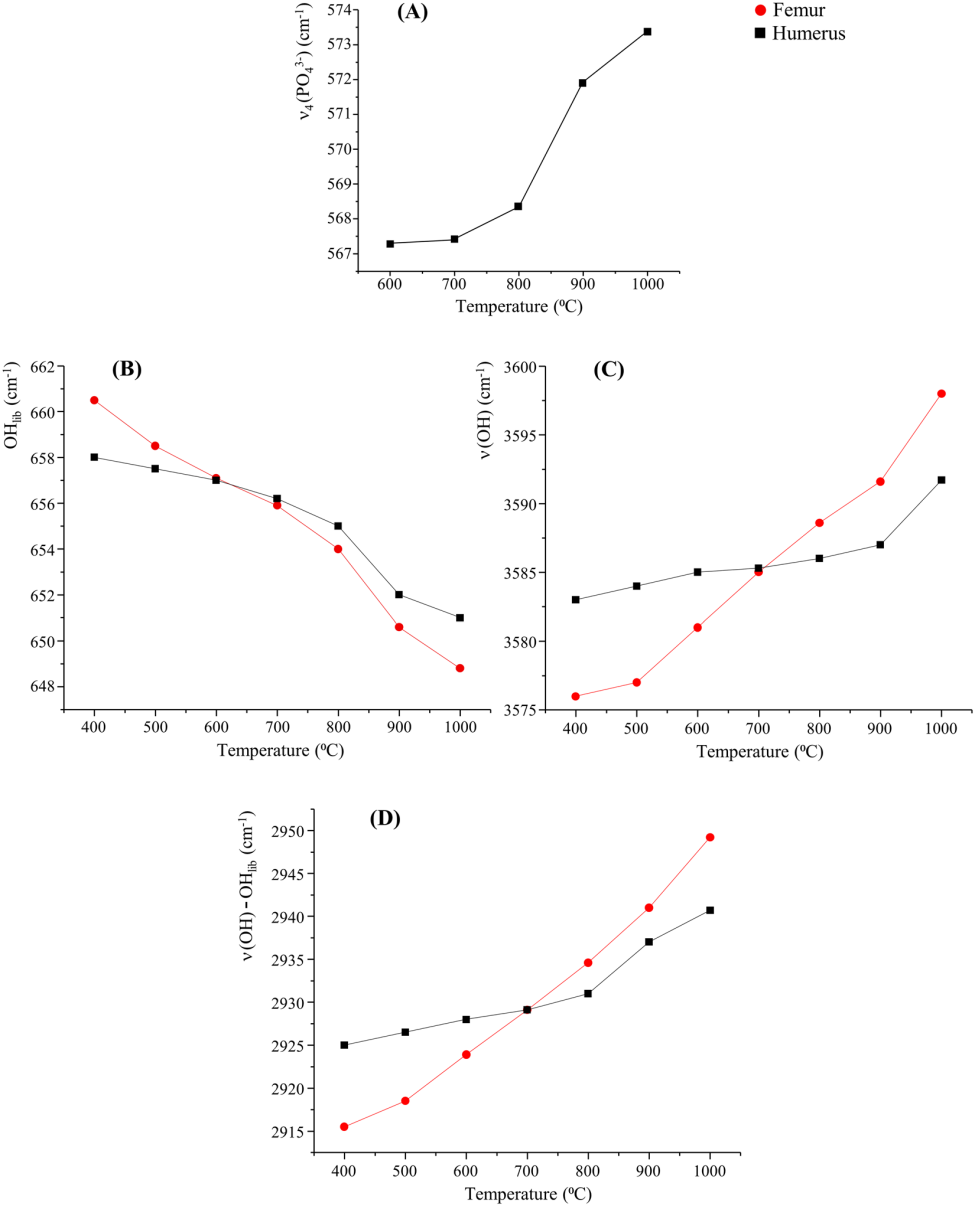
Temperature dependence of INS vibrational wavenumbers measured for human femur and humerus burned at different temperatures (400 to 1000 °C): (**A**) ν_4_(PO_4_^3−^); (**B**) (OH)_lib_; (**C**) ν(OH); (**D**) ν(OH)-(OH)_lib_.

A noticeable temperature dependence was found for several INS features detected for the samples under study: (i) for the lowest energy ν_4_(PO_4_^3−^) signal (Fig. 6(A)), its frequency decreasing (by up to *ca*. 6 cm^−1^) for lower temperatures, until it becomes indistinguishable below 600 °C; (ii) for bioapatite’s OH librational and stretching signals, that are progressively separated as temperature increases (the former shifting to lower frequencies while the latter deviates to the blue) (Fig. 6(B–D)). These quantitative relationships between particular spectral bands and heating temperature are clear evidence of rearrangements in the hydrogen-bond pattern within the hydroxyapatite network, concurrent with a crystallinity change during the burning process^32,53^. Interestingly, this effect was not identical for both types of bone presently investigated (femur and humerus), as reported for previous measurements on burned human bone^36^, being more significant for femur regarding the OH signals (Fig. 6). This observation seems to imply that the effect of burning does not completely obliterate the differences in the skeletal samples prior to burning and needs further investigation. The signals undergoing defined wavenumber deviations upon burning may be taken as reliable biomarkers (proxies) of heat-prompted microcrystallinity rearrangements (Table 2). Their unequivocal assignment and relationship to FTIR-ATR data – quite straightforward for these infrared active OH_lib_, OH_stretch_ and ν_4_(PO_4_^3−^) bands – will enable a routine analysis of burned bone samples through this easy-to-use, benchtop vibrational technique.

**Figure 7:**
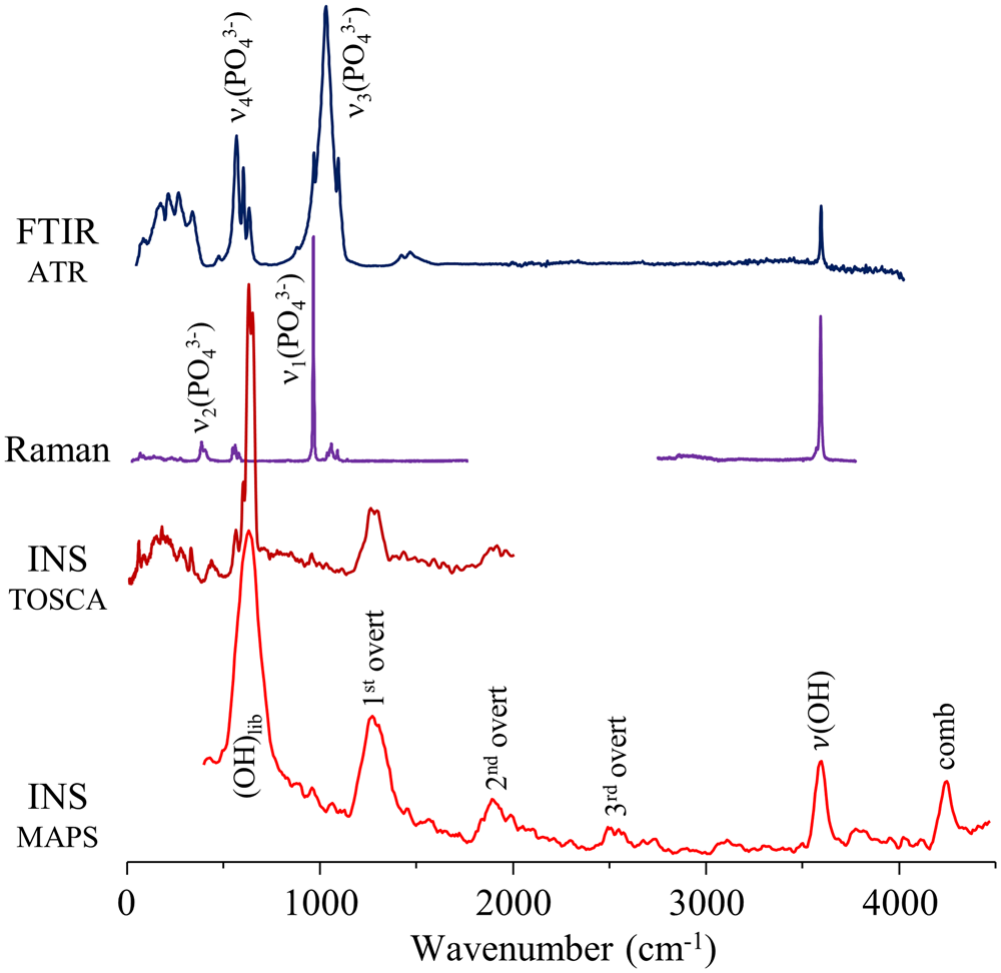
Vibrational profile – FTIR-ATR, Raman and INS (measured in TOSCA and MAPS) – of human femur burned at 1000 °C.

Figure 7 represents the whole vibrational profile of human femur burned at 1000 °C, obtained by FTIR-ATR, Raman and INS (measured in both MAPS and TOSCA), as an example of the complementary information only accessible for these types of samples through the combined use of several vibrational techniques. While the ν_3_^as^(PO_4_^3−^) and ν_3_(CO_3_^2−^)_B/A_ modes are clearly detected in the infrared, Raman allows us to observe ν_1_(PO_4_^3−^), ν_4_(PO_4_^3−^), ν_2_(PO_4_^3−^) and ν_1_(CO_3_^2−^)_B_, and INS yields the low frequency lattice modes of bone’s inorganic matrix as well as hydroxyapatite’s OH libration and stretching (detected simultaneously with very high sensitivity).

**Figure 8:**
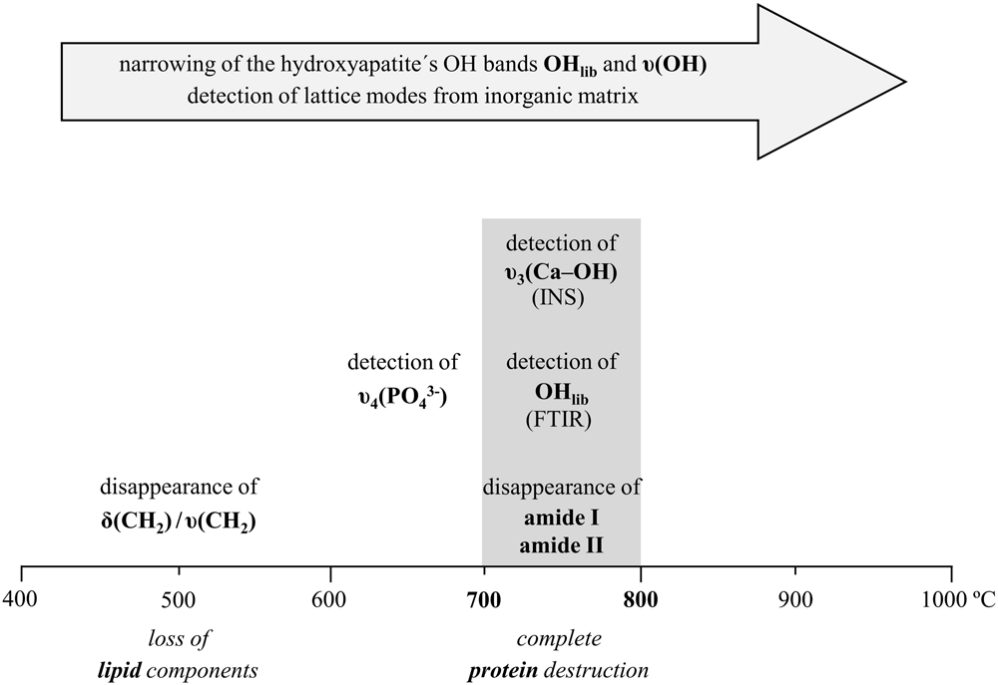
Schematic representation of the main spectral changes observed upon bone combustion (from 400 to 1000 °C).

Throughout the burning process, the most significant spectral differences were observed between 700 and 800 °C and summarized in Fig. 8: (i) complete loss of the protein constituents (disappearance of amide I and II infrared bands, Fig. 2); (ii) significant narrowing of hydroxyapatite’s OH_lib_ and υ(OH) INS signals (Figs 4 and 5(A)), and appearance of the OH_lib_ infrared (Fig. 2); (iii) more defined lattice modes from the inorganic matrix, detected in far-IR and INS – namely (Ca–PO_4_), OH_translation_, and ν_2_(HPO_4_^2−^)/ν_2_(PO_4_^3−^) (Figs 2 and 5(B)); (iv) appearance of the ν_3_(Ca–OH) INS feature (upon loss of type A carbonates) (Fig. 5(B)).

## Conclusions

The combined INS, Raman and infrared results presented here have enabled a thorough interpretation and validation of the existing vibrational spectroscopy data for skeletal human remains (including FTIR-derived indexes). Inelastic neutron scattering spectroscopy, in particular, led to the clarification of some unsettled issues such as: (i) the low energy vibrational pattern of bone subject to different temperatures; (ii) the temperature dependence of some characteristic lattice vibrational modes, and its relationship to hydroxyapatite’s crystallinity; (iii) the presence of HPO_4_^2−^; (iv) the state of hydroxylation of bone’s inorganic lattice; (v) the carbonate by hydroxyl substitution within bioapatite’s matrix upon increasing temperatures (associated to the crystallite size). In addition, the combined MAPS and TOSCA experiments yielded a consistent set of results that were crucial for attaining an accurate assignment of the FTIR and Raman spectra (measured for the same samples), leading to the identification of reliable biomarkers (proxies) of heat-elicited diagenesis, namely OH_lib_, ν(OH) and ν_4_(PO_4_^3−^) bands, for which a distinctive quantitative temperature dependence was obtained.

This validates the analysis of burned skeletal remains by benchtop vibrational techniques such as FTIR-ATR, which is a low-cost, non-destructive and rapid method requiring very small amounts of bone and no sample preparation. The spectroscopic biomarkers of bone heat-induced diagenesis will be linked to pre-burned bone dimensions, finally providing a definitive correlation between crystalline structure and heat-prompted macroscopic dimensional changes. This should have an immediate impact on bone analysis, establishing FTIR-ATR as a routine technique for characterizing and identifying burned skeletal remains.

The innovative methodology reported presently as a way of understanding human bone tissue subject to high temperatures is expected to pave the way for the study of other types of skeletal materials (*e*.*g*. charred bone and teeth), leading to an improved understanding of heat-induced diagenetic processes. Such a quantitative method for assessing dimensional changes in burned human bones will be of the utmost significance in forensic, anthropological and archaeological sciences, namely for the analysis of burned bones found in crime scenes (*e*.*g*. victim identification) or in archaeological settings.

## Materials and Methods

### Chemicals and Materials

The bone samples, from the Laboratory of Forensic Anthropology of the University of Coimbra, were collected from an unidentified human skeleton from the cemetery of Capuchos (Santarém, Portugal)^54^. The exact inhumation period of this skeleton is unknown, but was at least 3 years. The current study was focused on two types of bones – femur and humerus – from the same skeleton, to avoid inter-skeleton variability^11^. These bones were already defleshed, *i*.*e*. completely devoid of both soft tissue and marrow.

Apart from the burned samples, data was also acquired for the same bones prior to burning (intact samples, ground), for comparison purposes. The sample preparation and controlled burning (400 to 1000 °C) are described in the Supporting Information. The highly crystalline sample of calcium hydroxyapatite (Ca_2_(PO_4_)_6_(OH)_2_, Ca/P = 1.67) from NIST (Gaithersburg, MA, USA)^55^ was used as a reference (crystallinity index = 7.91, as compared to 3.79 for poorly crystalline commercial HAp).

#### Sample Preparation and Controlled Bone Burning

Femoral and humeral diaphyses were analysed (Fig. S1). After cutting each bone section (with a Dremel mini-saw electric tool), contaminants from the outer layer were removed by gentle sanding. For infrared and INS analysis, no further cleaning was needed^32^. Extensive handling was avoided, processes such as dehydration, defatting and deproteination being unnecessary for burned bones. Several adjacent bone slices were cut for each type of bone.

These bone fragments were subject to burning in an electric oven (Barracha K-3 three-phased 14 A), under controlled laboratory conditions regarding the intensity and duration of burning: temperatures in the range 400 to 1000 °C, at a heating rate *ca*. 6–10 °C/min, for 120 min. The burning process was performed in aerobic conditions (combustion). After burning, the bones were ground, followed by sieving (mesh size of 400 μm), yielding *ca*. 10 g of each sample.

Apart from the burned samples, data was also acquired for the same bones prior to burning (intact samples, ground), for comparison purposes.

### Fourier Transform Infrared Spectroscopy

The FTIR-ATR spectra were acquired for the powdered bone samples using Bruker Optics Vertex 70 FTIR spectrometers purged by CO_2_-free dry air and Bruker Platinum ATR single reflection diamond accessories. In the QFM-UC laboratory, a Ge on KBr substrate beamsplitter with a liquid nitrogen-cooled wide band mercury cadmium telluride (MCT) detector for the mid-IR interval (400–4000 cm^−1^), and a Si beamsplitter with a room temperature deuterated L-alanine doped triglycine sulfate (DLaTGS) detector with a polyethylene window for the far-IR range (50–600 cm^−1^), were used. At the ISIS Facility, a wide range MIR-FIR beamsplitter and a room temperature DLaTGS wide range detector were used (50–4000 cm^−1^).

Each spectrum was the sum of 128 scans, at 2 cm^−1^ resolution, and the 3-term Blackman–Harris apodization function was applied. Under these conditions, the wavenumber accuracy was better than 1 cm^−1^. The spectra were corrected for the frequency dependence of the penetration depth of the electric field in ATR (considering a mean reflection index of 1.25) using the Opus 7.2 spectroscopy software.

### Raman Spectroscopy

Raman spectra of the powdered bone samples (in the 100–1800 cm^−1^ and 2750–3500 cm^−1^ intervals) were recorded using a Horiba Jobin-Yvon T64000 spectrometer in direct configuration mode (focal distance 0.640 m, aperture f/7.5), equipped with a holographic grating of 1800 grooves∙mm^−1^. The entrance slit was set to 200 μm. Rayleigh elastic scattering was rejected by a notch filter, which reduces its intensity by a factor of 10^6^. The detection system was a liquid nitrogen cooled non-intensified 1024 × 256 pixels (1”) CCD camera. The 514.5 nm line of an Ar^+^ laser (Coherent, model Innova 300-05) was used as the excitation radiation, yielding ca. 10 mW at the sample position. All the spectra were recorded using an Olympus 50x objective (MSPlan 50, infinity corrected, NA 0.80, WD 0.47 mm).

All spectra were recorded with 5 accumulations and 30 seconds of exposure, at <1 cm^−1^ spectral resolution, at room temperature with the samples placed onto glass microscope slides.

### Inelastic Neutron Scattering Spectroscopy

The INS spectra were obtained at the ISIS Pulsed Neutron and Muon Source of the STFC Rutherford Appleton Laboratory (United Kingdom), using the time-of-flight, high resolution broad range spectrometers MAPS^56,57^ and TOSCA^57–59^.

In MAPS, three incident energies were used (968, 2024 and 5240 cm^−1^) in order to accurately observe all the bands from bioapatite, in both the low and high frequency ranges – namely the OH libration, its overtones and the OH stretch mode.

The samples (4–10 g) were wrapped in aluminium foil and fixed onto 4 × 4 cm thin walled aluminium cans. To reduce the impact of the Debye-Waller factor (the exponential term in Equation (1)) on the observed spectral intensity, the samples were cooled to 5–10 K. Data were recorded in the energy range 0 to 6000 cm^−1^ (MAPS) and 0 to 4000 cm^−1^ (TOSCA), and converted to the conventional scattering law, S(Q, ν) *vs* energy transfer (in cm^−1^) using the MANTID program (version 3.4.0)^60^.

## Electronic supplementary material

Supplementary information

## Data Availability

The datasets generated during and/or analysed during the current study are available from the corresponding author upon reasonable request.
