# Supplementary material for: Heat-induced Bone Diagenesis Probed by Vibrational Spectroscopy
Source: Sci Rep. 2018 Oct 29;8:15935. doi: 10.1038/s41598-018-34376-w (PMC6206023; doi:10.1038/s41598-018-34376-w)
Supplement: Supplementary file 1 — Supplementary information [file 41598_2018_34376_MOESM1_ESM.pdf]

Heat-induced Bone Diagenesis Probed by Vibrational Spectroscopy

M.P.M. Marques[a,b], A.P. Mamede[a], A.R. Vassalo[a,c,d], C. Makhoul[a,c], E. Cunha[b,c], D. Gonçalves[c,d,e], S.F. Parker[f] and L.A.E. Batista de Carvalho[a]*

[a]"Molecular Physical Chemistry" R&D Unit, Department of Chemistry, University of Coimbra, Portugal
[b]Department of Life Sciences, University of Coimbra, Portugal
[c]Lab. Forensic Anthropology, Centre for Functional Ecology, University of Coimbra, Portugal
[d]Research Centre for Anthropology and Health (CIAS), University of Coimbra, Portugal
[e]Archaeosciences Lab., Directorate General Cultural Heritage (LARC/CIBIO/InBIO), Lisbon, Portugal
[f]ISIS Facility, STFC Rutherford Appleton Laboratory, Chilton, Didcot, OX 11 0QX, United Kingdom

Supplementary Information

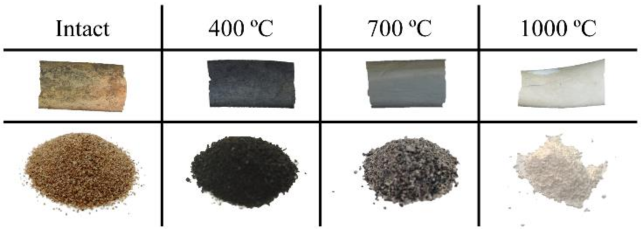

Figure S1. Sections from human femoral and humeral diaphysis, analyzed in the present work (before and after grinding): intact, and burned at 400, 700 and 1000 ºC.
